# Impact of food insecurity on cognitive health in older adults: insights from the NHANES 2011–2014 data

**DOI:** 10.3389/fnut.2024.1421970

**Published:** 2024-07-03

**Authors:** Yanwei Zhang, JiaWen Jiang, Dekun Yin

**Affiliations:** ^1^Department of Anesthesiology, Shanghai Ninth People’s Hospital, Shanghai Jiao Tong University School of Medicine, Shanghai, China; ^2^School of Health Science and Engineering, University of Shanghai for Science and Technology, Shanghai, China; ^3^Department of Anesthesiology, Funing People’s Hospital of Jiangsu, Yancheng, Jiangsu, China

**Keywords:** food insecurity, cognitive decline, elderly health, NHANES, nutrition

## Abstract

**Purpose:**

To investigate the relationship between food insecurity and cognitive decline among elderly Americans.

**Methods:**

Utilizing NHANES 2011–2014 data, we examined cognitive function via the Immediate Recall Test (IRT), Delayed Recall Test (DRT), Animal Fluency Test (AFT), Digit Symbol Substitution Test (DSST) and assessed food security through the US Food Security Survey Module. Multiple regression models were used to adjust for demographic and health variables.

**Results:**

Food insecurity demonstrated a significant association with lower cognitive function scores. The effects of food insecurity on cognitive function were moderated by factors such as smoking and alcohol use, indicating a direct influence of food insecurity on cognitive decline.

**Conclusion:**

This study underscores the importance of food security for cognitive health in the elderly and advocates for targeted interventions to address nutritional disparities and enhance cognitive functioning in aging populations.

## 1 Introduction

Cognition involves the process of acquiring and applying knowledge, which includes information processing, and is a fundamental human mental activity (1). This process encompasses sensation, perception, memory, thought, imagination, and language. Cognitive impairment covers a broad spectrum of neurological disorders that significantly affect memory, thinking, and behavior, diminishing an individual’s capacity to perform daily activities and sustain independent living (2). The US Department of Health and Human Services reports that over one-fifth of the US population will be aged 65 or older by 2030 (3). With an aging population, the incidence of cognitive impairment and dementia is projected to rise substantially. Currently, about 5.8 million older Americans are diagnosed with Alzheimer’s disease, with this figure expected to escalate to nearly 14 million by 2050 (4). Cognitive decline not only compromises the quality of life of affected individuals but also imposes a significant burden on families and the social healthcare system. Consequently, cognitive health has emerged as a crucial public health concern for the aging US population (5, 6).

Food security is defined as the access to adequate, safe, and nutrient-rich food consistently and universally, fulfilling physiological needs and food preferences to sustain an active and healthy lifestyle (7). Despite the stable food supply enjoyed by the majority of the US population, food insecurity persists, particularly among low-income families. According to the US Department of Agriculture, approximately 10.3 million households (7.8% of total households) faced challenges in food access in 2020 (8). The link between food security and cognitive health is well-documented. Numerous studies have demonstrated that a lack of proper nutrition is associated with compromised cognitive function (9–11). Undernutrition may exacerbate cognitive decline, especially in older adults whose cognitive health is already vulnerable (12, 13). Nutrients such as omega-3 fatty acids, antioxidants, and vitamins play critical roles in maintaining brain health and protecting against cognitive decline (14–16). For instance, healthy dietary patterns like the Mediterranean diet, which is rich in fruits, vegetables, and whole grains, have been shown to have neuroprotective effects and are associated with better cognitive outcomes in older adults (17–20). Conversely, food insecurity can lead to poor dietary choices, resulting in insufficient intake of essential nutrients that support cognitive function (21).

This study aims to address these gaps by utilizing a robust dataset from the National Health and Nutrition Examination Survey (NHANES), which offers detailed and diverse demographic and health behavior data. This approach allows for a more nuanced exploration of the relationship between different levels of food security and cognitive function among older adults in the US, distinguishing this work from prior research by providing clearer insights into potential causal relationships.

## 2 Materials and methods

### 2.1 Study population

**FIGURE 1:**
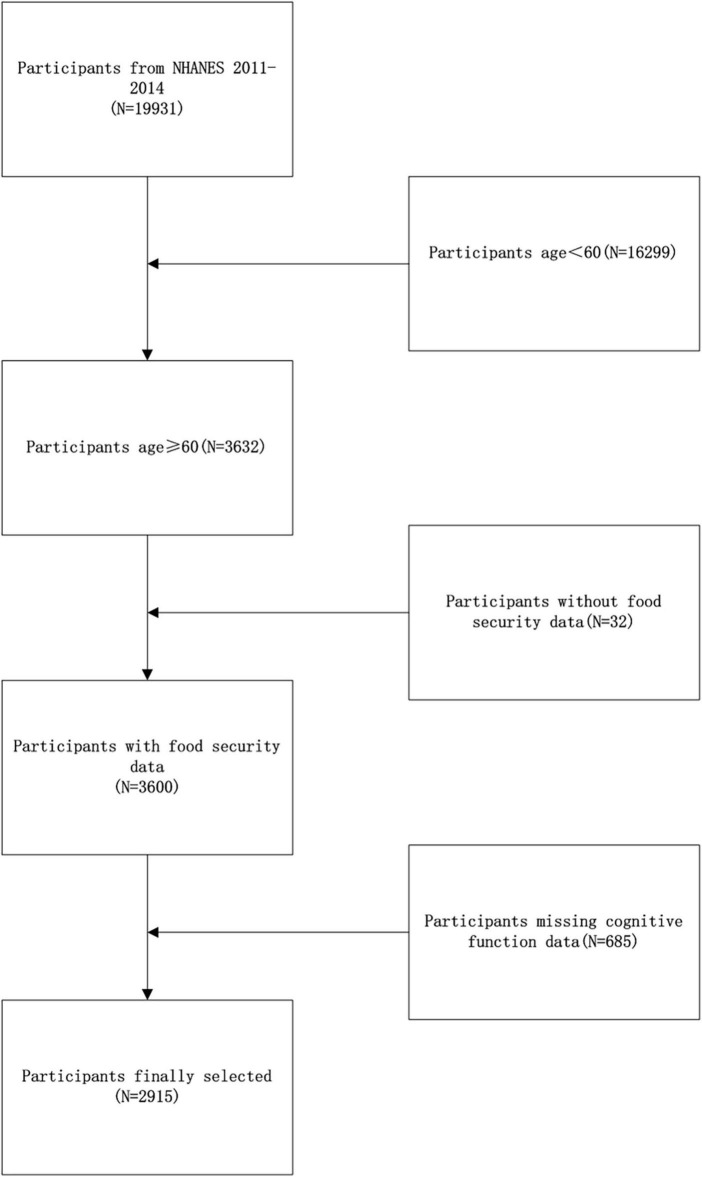
Flow chart showing the NHANES participants’ selection.

The National Health and Nutrition Examination Survey (NHANES) is a cross-sectional survey that aims to gather detailed data on the health and nutritional status of the American populace. This survey employs a sophisticated stratified, multistage probability sampling design, focusing specifically on the ambulatory population. Conducted by the National Center for Health Statistics (NCHS), the survey protocol received approval from its ethical review board, and all participants provided written informed consent. For this study, the NHANES dataset covering the period from 2011 to 2014 was selected, as it uniquely incorporates measures of cognitive function for older adults aged 60 and above. Initially, the survey screened 19,931 participants. However, due to participants age < 60 (16266), missing food security data (32) and complete cognitive function data (685), the final analysis included only 2,915 participants (as shown in Figure 1).

### 2.2 Food security diagnosis

The assessment of household food security in the United States utilized the US Food Security Survey Module (FSSM), which is recognized as the definitive standard for measuring food insecurity nationwide. This validated tool comprises ten questions that an adult household member answers to reflect the food security situation over the past twelve months. The NHANES documentation outlines the classification of food security status based on the count of affirmative responses in the adult module: zero affirmatives indicate full food security, one to two suggest marginal food security, three to five signify low food security, and six to ten affirmatives represent very low food security. Aligning with established research, the study aggregated the categories of full and marginal food security into a single “food security” group, and combined the low and very low categories into a “food insecurity” group (22).

### 2.3 Measurement of cognitive function

In neuropsychological evaluations, tests such as the Immediate Recall Test (IRT), Delayed Recall Test (DRT), Animal Fluency Test (AFT), and Digit Symbol Substitution Test (DSST) are crucial for assessing cognitive functions. This study utilized the Word Learning and Recall modules from the Consortium to Establish a Registry for Alzheimer’s Disease (CERAD), including both the IRT and DRT. These assessments are essential for evaluating memory capabilities and diagnosing cognitive impairments and dementia. The CERAD Word Learning subtest (CERAD W-L) requires participants to recall ten unrelated words immediately across three trials. After a brief distractive task, a delayed recall phase is conducted to assess both short-term and long-term memory retention. To measure verbal fluency and executive functions, the AFT instructs participants to name as many animals as possible within one minute, assessing speed and dexterity in vocabulary retrieval, often indicative of prefrontal lobe impairment. The DSST focuses on attention, processing speed, working memory, learning, and hand-eye coordination, as participants match symbols with numbers within two minutes. These tests are vital for diagnosing Alzheimer’s disease, mild cognitive impairment (MCI), and other dementias, offering critical insights for developing appropriate treatment and intervention strategies.

### 2.4 Covariates

In this research, we considered a comprehensive range of covariates: age, gender, race, education level, body mass index (BMI), poverty-income ratio (PIR), presence of cardiovascular disease (CVD), smoking history, frequency of alcohol consumption, diabetes, and hypertension. All covariate data were obtained from the publicly available datasets of the National Health and Nutrition Examination Survey (NHANES), which provides extensive resources for research utilization.

### 2.5 Statistical analysis

Statistical analyses in this study were carried out using EmpowerStats software (version 2.0) and the R statistical package (version 4.2) to ensure a robust analytical approach. Sampling weights from NHANES were applied to all analyses to enhance representativeness. Missing data among covariates were handled through interpolation. The participants were divided into two groups based on their food security status. Differences in demographic characteristics between these groups were analyzed using chi-square tests for categorical variables and *t*-tests for continuous variables. For inferential analysis, multifactor linear regression models were employed to examine the relationship between food insecurity and cognitive function, adjusting for the covariates mentioned. This approach was chosen to control for potential confounders that might influence the relationship between food security status and cognitive outcomes. Model 1 was unadjusted, Model 2 adjusted for demographic factors (age, gender, race), and Model 3 was fully adjusted for all listed covariates. Additionally, stratified analyses and interaction tests were conducted to probe the dynamics of this relationship across various demographic segments. Statistical significance was defined at a two-tailed *p*-value of less than 0.05.

### 2.6 Ethical considerations

The survey protocol, including data collection and analysis, received approval from the National Center for Health Statistics (NCHS) ethical review board. Informed consent was obtained from all participants before data collection.

## 3 Results

### 3.1 Baseline characteristics

Table 1 illustrates the demographic characteristics of 2,915 older Americans, each at least 60 years old, categorized into two groups based on food security. The average age of participants was 69.22 ± 6.65 years. The sample comprised 45.53% males and 54.47% females. The food security group included 2,558 individuals, accounting for 87.75% of the total sample, while the food insecurity group comprised 357 individuals, or 12.25%. Comparative analysis revealed that the food insecurity group had a lower mean age, a smaller proportion of non-Hispanic whites, a higher body mass index (BMI), a lower poverty-income ratio (PIR), and lesser educational attainment. Additionally, this group showed a higher percentage of current smokers, a lower frequency of alcohol consumption, and a higher prevalence of diabetes mellitus, hypertension, and cardiovascular disease (CVD).

**TABLE 1:** Weighted characteristics of the study population based on food security.

Characteristics	All (*n* = 2,915)	Food security		*P*-value
		Yes (*n* = 2,558)	No (*n* = 357)	
Age (years)	69.22 ± 6.65	69.39 ± 6.68	66.97 ± 5.89	< 0.0001
Gender (%)				0.0512
Male	45.53	46.03	39.31	
Female	54.47	53.97	60.69	
BMI	29.05 ± 6.23	28.89 ± 6.05	30.98 ± 7.95	< 0.0001
PIR	3.06 ± 1.55	3.18 ± 1.52	1.46 ± 0.98	< 0.0001
RACE (%)				< 0.0001
Mexican American	3.35	2.79	10.37	
Other Hispanic	3.66	2.99	12.13	
Non-Hispanic White	79.61	81.54	55.31	
Non-Hispanic Black	8.4	7.62	18.24	
Non-Hispanic Asian	3.27	3.38	1.91	
Other Race	1.71	1.69	2.05	
Education (%)				< 0.0001
< high school	5.71	4.61	19.48	
9–11th grade	10.28	9.32	22.46	
High school graduate	22.19	21.58	29.78	
Some college or AA degree	31.47	32.29	21.25	
College graduate or above	30.35	32.2	7.03	
Smoked ≥ 100 cigarettes (%)				0.0382
Yes	50.43	49.93	56.67	
No	49.57	50.07	43.33	
Alcohol intakes ≥ 12 drinks/year (%)				0.0348
Yes	73.07	73.58	66.68	
No	26.93	26.42	33.32	
High blood pressure (%)				< 0.0001
Yes	59.14	57.98	73.72	
No	40.86	42.02	26.28	
Diabetes (%)				< 0.0001
Yes	19.36	18.37	31.84	
No	76.57	77.71	62.21	
Boardline	4.07	3.93	5.95	
CVD				< 0.0001
Yes	24.3	23.41	35.53	
No	75.7	76.59	64.47	
IRT	19.71 ± 4.49	19.84 ± 4.44	18.09 ± 4.77	< 0.0001
DRT	6.22 ± 2.30	6.27 ± 2.29	5.66 ± 2.33	0.0002
AFT	18.07 ± 5.69	18.23 ± 5.70	16.00 ± 5.13	< 0.0001
DSST	51.84 ± 16.93	52.77 ± 16.54	40.21 ± 17.45	< 0.0001

Mean ± SD for continuous variables: the *P*-value was calculated by the weighted linear regression model; (%) for categorical variables: the *P*-value was calculated by the weighted chi-square test.

### 3.2 Relationship between food security and cognitive function

Table 2 presents results from multifactorial regression analyses examining the relationship between food insecurity and cognitive functioning, specifically focusing on Immediate Recall Test (IRT), Delayed Recall Test (DRT), Animal Fluency Test (AFT), and Digit Symbol Substitution Test (DSST). In Model 3, which adjusts all covariates, food insecurity significantly reduced IRT scores (β = −0.94; 95% CI: −1.54, −0.34; *P* = 0.0021). Additionally, the analysis using the US Food Safety Surveillance Metric (FSSM) revealed significant associations between different levels of food security and IRT scores: marginal food security (β = −0.23; *P* = 0.5204), low food security (β = −0.91; *P* = 0.0154), and very low food security (β = −1.07; *P* = 0.0220).

**TABLE 2:** Associations between food security and DSST.

Exposure	Model 1 β (95% CI) *P*-value	Model 2 β (95% CI) *P*-value	Model 3 β (95% CI) *P*-value
**IRT**			
Food security	Ref	Ref	Ref
Food insecurity	−1.75 (−2.37, −1.13) < 0.0001	−1.97 (−2.55, −1.38) < 0.0001	−0.94 (−1.54, −0.34) 0.0021
**FSSM**			
Full food security	Ref	Ref	Ref
Marginal food security	−0.95 (−1.69, −0.20) 0.0128	−0.89 (−1.59, −0.20) 0.0113	−0.23 (−0.92, 0.47) 0.5204
Low food security	−1.84 (−2.62, −1.05) < 0.0001	−1.95 (−2.68, −1.22) < 0.0001	−0.91 (−1.65, −0.17) 0.0154
Very low food security	−1.74 (−2.72, −0.76) 0.0005	−2.18 (−3.09, −1.27) < 0.0001	−1.07 (−1.99, −0.15) 0.0220
**DRT**			
Food security	Ref	Ref	Ref
Food insecurity	−0.61 (−0.93, −0.29) 0.0002	−0.74 (−1.04, −0.44) < 0.0001	−0.37 (−0.68, −0.06) 0.0209
**FSSM**			
Full food security	Ref	Ref	Ref
Marginal food security	−0.28 (−0.67, 0.10) 0.1485	−0.25 (−0.61, 0.10) 0.1661	−0.03 (−0.40, 0.33) 0.8519
Low food security	−0.47 (−0.88, −0.07) 0.0205	−0.55 (−0.93, −0.17) 0.0045	−0.18 (−0.57, 0.21) 0.3605
Very low food security	−0.87 (−1.37, −0.37) 0.0007	−1.11 (−1.57, −0.64) < 0.0001	−0.69 (−1.17, −0.21) 0.0047
**AFT**			
Food security	Ref	Ref	Ref
Food insecurity	−2.23 (−3.02, −1.45) < 0.0001	−2.04 (−2.78, −1.30) < 0.0001	−0.39 (−1.13, 0.34) 0.2938
**FSSM**			
Full food security	Ref	Ref	Ref
Marginal food security	−2.41 (−3.35, −1.47) < 0.0001	−1.79 (−2.66, −0.92) < 0.0001	−0.75 (−1.60, 0.10) 0.0821
Low food security	−2.57 (−3.56, −1.59) < 0.0001	−2.10 (−3.03, −1.18) < 0.0001	−0.42 (−1.33, 0.48) 0.3570
Very low food security	−2.03 (−3.26, −0.79) 0.0013	−2.31 (−3.46, −1.17) < 0.0001	−0.61 (−1.73, 0.51) 0.2856
**DSST**			
Food security	Ref	Ref	Ref
Food insecurity	−12.15 (−14.44, −9.85) < 0.0001	−10.84 (−12.79, −8.88) < 0.0001	−3.83 (−5.65, −2.01) < 0.0001
**FSSM**			
Full food security	Ref	Ref	Ref
Marginal food security	−10.88 (−13.61, −8.16) < 0.0001	−8.52 (−10.81, −6.24) < 0.0001	−3.70 (−5.80, −1.60) 0.0006
Low food security	−13.54 (−16.40, −10.68) < 0.0001	−11.34 (−13.76, −8.92) < 0.0001	−4.48 (−6.72, −2.24) < 0.0001
Very low food security	−11.45 (−15.03, −7.86) < 0.0001	−11.77 (−14.77, −8.76) < 0.0001	−4.09 (−6.86, −1.32) 0.0038

Model 1: Variables were not adjusted. Model 2: Adjustments were made to age, gender, and race. Model 3: Age, gender, race, BMI, PIR, HBP, diabetes, CVD, smoking status, and alcohol intake were adjusted.

For the Delayed Recall Test (DRT), Model 3 showed that food insecurity continued to significantly lower DRT scores (β = −0.37; 95% CI: −0.68, −0.06; *P* = 0.0209). The FSSM analysis showed marginal food security (β = −0.03; *P* = 0.8519), low food security (β = −0.18; *P* = 0.3605), and very low food security (β = −0.69; *P* = 0.0047).

In the Animal Fluency Test (AFT), Model 3 indicated that the association between food insecurity and AFT scores was not statistically significant (β = −0.39; 95% CI: −1.13, 0.34; *P* = 0.2938). However, the FSSM analysis indicated marginal food security (β = −0.75; *P* = 0.0821), low food security (β = −0.42; *P* = 0.3570), and very low food security (β = −0.61; *P* = 0.2856).

For the Digit Symbol Substitution Test (DSST), Model 3 revealed that food insecurity significantly reduced DSST scores (β = −3.83; 95% CI: −5.65, −2.01; *P* < 0.0001). The FSSM analysis showed marginal food security (β = −3.70; *P* < 0.05), low food security (β = −4.48; *P* < 0.05), and very low food security (β = −4.09; *P* < 0.05).

These analyses consistently demonstrate that food insecurity is significantly associated with poorer performance across various cognitive function tests. This relationship remains robust even after controlling for multiple covariates, particularly in IRT, DRT, and DSST. The findings emphasize the critical need for interventions addressing food insecurity to mitigate its adverse effects on cognitive health.

### 3.3 Stratified and interaction analysis

Supplementary Table 1 displays the outcomes of the stratified analyses and interaction tests designed to investigate potential factors that may affect the relationship between food security and performance on cognitive function. The results indicate that both smoking and alcohol consumption may modify this association between food insecurity and IRT, DSST, with a more pronounced negative correlation observed. Importantly, despite these effects, the direction of the effect estimates remained consistent across most examined subgroups (except for participants test AFT with BMI ≤ 18.5), and no additional significant interactions were detected.

## 4 Discussion

This study investigated 2,915 older Americans, each at least 60 years of age, to examine the correlation between food insecurity and cognitive function. It was observed that individuals in the food insecurity group were younger on average, had a lower percentage of non-Hispanic whites, higher BMI, lower poverty-to-income ratios (PIRs), lesser educational attainment, a higher percentage of current smokers, reduced alcohol consumption, and a higher prevalence of diabetes, hypertension, and cardiovascular disease (CVD). A significant negative association between food insecurity and IRT, DRT, DSST scores was identified and as the level of food insecurity increases, the decline in cognitive function becomes more pronounced. Further, results from interaction tests suggested that smoking and alcohol consumption might have modified the relationship between food security and cognitive function. These findings address a research gap and highlight the profound impact of food insecurity on cognitive health.

Several studies underscore the link between healthy dietary habits and cognitive protection. For instance, a study in Madrid reported that older adults consuming a rich and nutritious diet exhibited superior cognitive test performance (23). Research from India indicated that food-secure older adults experienced fewer issues with memory and numeracy, underscoring the critical role of food security in cognitive maintenance (24). A study in Massachusetts revealed that very low levels of food security were prevalent and correlated with reduced cognitive performance (25). Furthermore, systematic assessment studies have demonstrated that food insecurity, whether experienced early or later in life, is linked to poorer cognitive abilities, suggesting that individuals facing food insecurity may be at an elevated risk of cognitive decline (26). These observations emphasize the necessity for policies and intervention strategies to mitigate food insecurity.

In the domain of food security and cognitive function among older adults, numerous studies have highlighted the connection between healthy eating habits and cognitive protection. Research in rural China, demonstrated that the healthy dietary pattern safeguarded older adults from cognitive decline, which was based on the consumption of rice and flour, red meat, chicken, vegetables, seafood, and fruits (27). Additionally, adherents to the Mediterranean and MIND dietary patterns exhibited superior cognitive performance, showcasing the neuroprotective properties of these diets in older adults (28). The Nordic Prudent Dietary Pattern (NPDP) has been linked to sustained cognitive function among Nordic older adults, with followers of this diet showing the lowest risk of cognitive decline and reductions in MMSE scores over a six-year span (29). These findings underscore the influence of regional and cultural dietary habits on cognitive preservation in older adults and highlight the importance of public health strategies that promote healthy dietary patterns, particularly through nutritional interventions targeting the elderly.

Food security and adequate nutritional support are crucial for maintaining and enhancing cognitive function in old age. A negative correlation exists between food insecurity and cognitive function, potentially due to a blend of biological and psychosocial factors. Food insecurity might lead to insufficient intake of critical nutrients (30) such as Omega-3 fatty acids, antioxidants (e.g., vitamins E and C), vitamin B complex, and minerals (e.g., zinc and iron), which are vital for nerve cell structure and function. For example, omega-3 fatty acids are essential for neuronal membrane integrity and function, and their deficiency has been linked to impaired cognitive function and increased risk of neurodegenerative diseases (31, 32). Antioxidants play a crucial role in protecting neurons from oxidative stress, which is a significant factor in the pathogenesis of cognitive decline and dementia (33, 34). Deficiencies in these nutrients can impair nerve conduction and disrupt signaling and memory formation processes in the brain (35–37). Chronic food insecurity can also trigger persistent psychological and physiological stress, leading to a chronic stress response (20, 21). This stress can compromise brain function by activating stress hormones like cortisol, which can be harmful to the hippocampus, a crucial area for memory storage in the brain (38, 39). This stress can compromise brain function by activating stress hormones like cortisol, which can be harmful to the hippocampus, a crucial area for memory storage in the brain (40–42). Additionally, poor diet and malnutrition may elevate inflammation levels in the body, which studies have shown to interfere with neural pathways in the brain, accelerating the progression of neurodegenerative diseases such as Alzheimer’s disease (37, 43). The upregulation of inflammatory cytokines, such as tumor necrosis factor alpha and interleukin 6, is a significant factor in cognitive decline. Moreover, food insecurity often correlates with lower socioeconomic status, which can restrict access to quality education and healthcare services, and increase psychosocial stress (41, 44). This stress is often mediated by the activation of the hypothalamic-pituitary-adrenal (HPA) axis, resulting in elevated levels of cortisol, a stress hormone (45). Chronic elevation of cortisol can damage the hippocampus, a brain region integral to learning and memory (26, 45). Individuals with lower socioeconomic status may be less likely to engage in cognitively stimulating activities, such as reading and meaningful social interactions, which are known to promote cognitive health (46, 47). Finally, food insecurity may encourage unhealthy lifestyle choices such as smoking, excessive alcohol consumption, and physical inactivity, which are linked to cognitive decline (26, 48). It can also lead to decreased sleep quality; sleep deprivation is known to impair the brain’s cleansing mechanisms, such as the functioning of the lymphatic system, further impacting cognitive function (49, 50).

The association between food insecurity and lower cognitive function scores was intensified by the presence of smoking and alcohol consumption in this study. When assessing the impact of food insecurity on cognitive functioning in older adults, it is crucial to consider the roles of smoking and alcohol consumption as contributing behaviors. Research indicates that these behaviors not only independently affect cognitive health but also amplify cognitive impairment within the context of food insecurity. Specifically, cigarette smoking has been linked to cognitive decline in older women, notably affecting performance on various cognitive tests (51). Smoking has well-documented negative effects on cognitive function. Nicotine and other components of tobacco adversely affect blood supply to the brain, reducing oxygen and nutrient delivery, which can impair the function and survival of neurons (52). Moreover, smoking is closely linked to chronic conditions such as hypertension and diabetes, which have also been shown to affect cognitive function (53). Smokers tend to have higher levels of inflammation, and these inflammatory markers are associated with cognitive decline (54). In the context of food insecurity, this inflammatory response may be exacerbated, as inadequate nutrition can weaken the immune system, making individuals more susceptible to infections and other health issues (55). Alcohol consumption, particularly excessive drinking, has similarly significant negative effects on the brain (56). Alcohol interferes with the balance of neurotransmitters and affects the structure and function of the brain (57). Chronic excessive alcohol consumption can lead to brain atrophy, particularly affecting areas involved in memory and cognition (57). Studies have shown that alcohol use in older adults is not only associated with cognitive decline but also increases the risk of dementia (58). Within the context of food insecurity, the negative effects of alcohol may be amplified because malnutrition further compromises brain health, making it more vulnerable to alcohol toxicity. Collectively, smoking and alcohol consumption are significant exacerbators of the adverse effects of food insecurity on cognitive impairment in older adults.

To deal with food insecurity, it is essential to engage various stakeholders, each playing a critical role in addressing the complex challenges of food insecurity. Policymakers are urged to implement subsidy programs that make healthy foods more affordable and accessible, particularly in underserved communities (59). Supporting agricultural initiatives that promote local and sustainable food production can also increase the availability of fresh produce (60). Additionally, increasing funding for community-based programs like Meals on Wheels can provide direct food assistance to the elderly, particularly those who are homebound or living in food deserts (9). Healthcare providers can contribute by integrating regular screenings for food insecurity into routine medical assessments for older adults (61). This would enable timely referrals to nutritional support services. Educating older adults about available food assistance programs can ensure they have the information needed to access these resources (62). Moreover, adopting a holistic approach that considers nutritional status as a key component of managing cognitive health is crucial (63). Community organizations play a pivotal role by forming partnerships with local food banks, supermarkets, and agricultural organizations to secure a reliable supply of healthy foods (64). By adopting these recommendations, stakeholders can work collaboratively to enhance the cognitive health and overall wellbeing of older adults. This integrated approach not only addresses the immediate needs related to food insecurity but also promotes long-term health behaviors that are essential for maintaining cognitive function in older populations.

The strength of this study lies in its integrated analysis of the relationship between food insecurity and cognitive functioning. This multifactorial approach enriches our understanding of the intricate factors that influence cognitive health in older adults. Second, this study explores the moderating effects of behavioral factors such as smoking and alcohol consumption on the relationship between food insecurity and cognitive function. The results indicate that smoking and alcohol consumption may exacerbate the negative impact of food insecurity on cognitive health, underscoring the importance of addressing these behaviors in public health interventions. However, a notable limitation of this study is its cross-sectional design, which precludes the establishment of clear causal relationships between food security status and cognitive changes. Additional unmentioned limitations include potential confounders that were not fully controlled for, such as socio-economic factors and prior health conditions, which may influence both food security and cognitive health. Addressing these limitations in future research will be crucial to fully understand the complexities of how food insecurity impacts cognitive functioning in older adults.

## 5 Conclusion

This study underscores the detrimental impact of food insecurity on cognitive function among older Americans. Our findings highlight the critical need to enhance food security as a means to improve cognitive health and overall quality of life in the elderly. They advocate for targeted public health interventions designed to address these issues. Future research should concentrate on longitudinal analyses to more comprehensively understand the dynamics of these relationships and inform the development of effective strategies.

## Data availability statement

Publicly available datasets were analyzed in this study. This data can be found here: https://wwwn.cdc.gov/nchs/nhanes/Default.aspx.

## Ethics statement

The studies involving humans were approved by the National Center for Health Statistics (NCHS) Research Ethics Review Board. The studies were conducted in accordance with the local legislation and institutional requirements. The participants provided their written informed consent to participate in this study.

## Author contributions

YZ: Conceptualization, Data curation, Formal analysis, Funding acquisition, Investigation, Methodology, Resources, Software, Writing – original draft. JJ: Project administration, Resources, Validation, Writing – original draft. DY: Project administration, Supervision, Validation, Visualization, Writing – review & editing.

## Supplementary material

Supplementary Table 1 Linear regression analysis to identify variables that modify the correlation between food insecurity and cognitive function.
